# Pityriasis Lichenoides et Varioliformis Acuta as a Complication of COVID-19 Infection

**DOI:** 10.3390/dermatopathology9030028

**Published:** 2022-07-05

**Authors:** Henriette De La Garza, Elie Saliba, Monica Rosales Santillan, Candice Brem, Neelam A. Vashi

**Affiliations:** 1 Department of Dermatology, Boston University School of Medicine, Boston, MA 02118, USA; hadelaga@bu.edu (H.D.L.G.); elie_saliba@brown.edu (E.S.); mrosales@bu.edu (M.R.S.); candice.brem@bmc.org (C.B.); 2 Department of Dermatology, Brown University, Providence, RI 02903, USA; 3 Boston Health Care System, U.S. Department of Veterans Affairs, Boston, MA 02130, USA

**Keywords:** pityriasis lichenoides, pityriasis lichenoides et varioliformis acuta, PLEVA, rash, azithromycin, CD30, COVID-19

## Abstract

Pityriasis lichenoides is an acute and/or chronic skin disease associated with recurrent erythematous papules that self-resolve. While its etiology is unknown, preceding viral infection may play a role. We present an atypical case of a 40-year-old woman with pityriasis lichenoides et varioliformis acuta as a complication of a COVID-19 infection.

## 1. Introduction

Pityriasis lichenoides consists of a group of inflammatory skin disorders that includes both pityriasis lichenoides et varioliformis acuta (PLEVA) and pityriasis lichenoides chronica (PLC) [1].

PLEVA manifests as an acute eruption of small, erythematous to brown macules that rapidly develop into papules with overlying hemorrhagic crust while PLC has a more gradual manifestation of smaller red to brown flat maculopapules with an overlying scale. Both PLEVA and PLC follow a relapsing course with long periods of remission [1,2,3]. While the etiology of pityriasis lichenoides is unknown, it has been postulated to be a response to foreign antigens such as infectious agents and drugs [2].

## 2. Case

A 40-year-old woman presented with a 2-month history of new pruritic lesions initially appearing on the trunk then spreading to the face and extremities. Her past medical history was significant for hypothyroidism treated with Levothyroxine. The patient did not report any drug allergies, medication changes, or similar signs in other family members. A review of systems was also negative for fever, chills, nausea, vomiting, or significant weight changes. She was initially prescribed a 60 mg prednisone taper over the course of 4 weeks and topical clobetasol ointment, but she did not note any improvement in her lesions.

**Figure 1:**
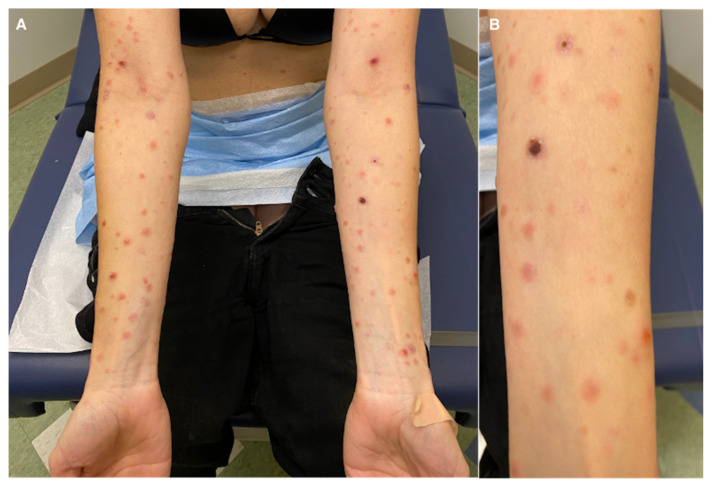
(**A**) Hemorrhagic crusting suggestive of PLEVA; (**B**) close-up.

On physical examination, there were several erythematous papules, some with overlying hemorrhagic crust or clear fluid vesicles, in different stages of healing located on the face, trunk, and upper and lower extremities. No mucosal findings were seen (Figure 1). Laboratory workup for HIV, syphilis, group A streptococcus, and infectious mononucleosis were negative. However, she stated that 2 weeks before the appearance of the skin lesions, she had a positive PCR COVID-19 test and a negative COVID vaccination history.

**Figure 2:**
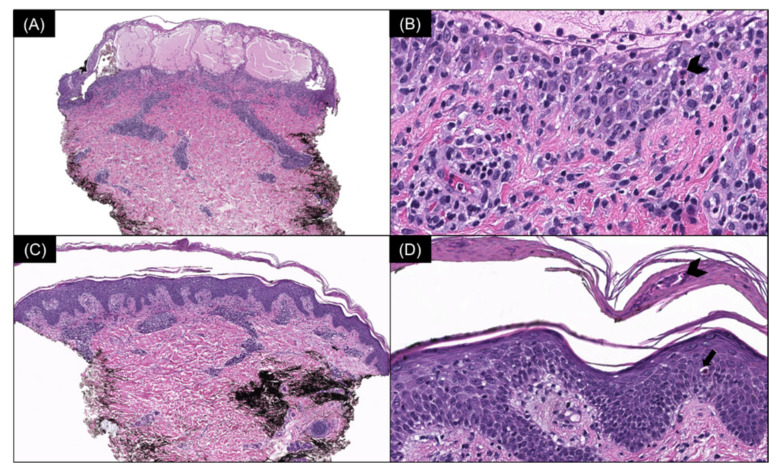
(**A**) Acute lesion: spongiotic vesiculation and reticular degeneration of the epidermis (H & E, 40×) with (**B**) intraepidermal necrotic keratinocytes (arrowhead) (H & E, 400×). (**C**) Chronic lesion: mild epidermal hyperplasia and a moderately dense superficial perivascular lymphocytic infiltrate (H & E, 40×) with (**D**) overlying parakeratosis containing neutrophils in the stratum corneum (arrowhead) and intraepidermal necrotic keratinocytes (arrow) (H & E, 200×).

**Figure 3:**
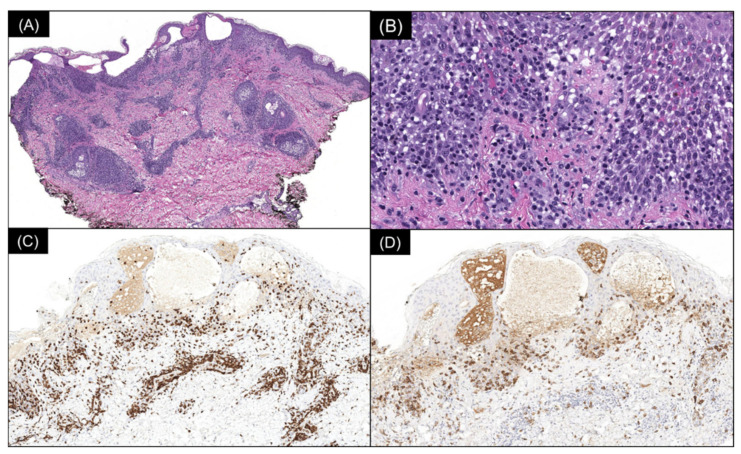
(**A**) Spongiotic vesiculation, reticular degeneration, and a wedge-shaped, moderately dense superficial perivascular lymphocytic infiltrate (H & E, 40×). (**B**) Higher power demonstrates basal layer vacuolization, lymphocytic exocytosis, and extravasated red blood cells (H & E, 200×). (**C**) Immunoperoxidase staining reveals the lymphocytic infiltrate to be composed of CD3 (+) T cells (80×) with (**D**) an intraepidermal population staining with CD30 (80×).

Multiple serial biopsies from both acute and chronic lesions were obtained. The acute lesions demonstrated spongiotic vesiculation and reticular degeneration (Figure 2A,B) while the chronic lesions exhibited more epidermal hyperplasia with overlying parakeratosis containing neutrophils in the stratum corneum (Figure 2C,D). Changes suggestive of PLEVA, such as necrotic keratinocytes, basal layer vacuolization, extravasated red blood cells, and lymphocytic exocytosis, were present in most biopsies (Figure 2 and Figure 3). The dermal infiltrate was predominantly composed of CD3 (+) T-lymphocytes admixed with rare CD20 (+) B-cells. In addition, a few of the biopsies also demonstrated scattered predominant intraepidermal CD30 (+) cells (Figure 3). A monoclonal TCR- γ gene rearrangement was also detected in one of the specimens. The histologic differential diagnosis included PLEVA and lymphomatoid papulosis (LyP).

**Figure 4:**
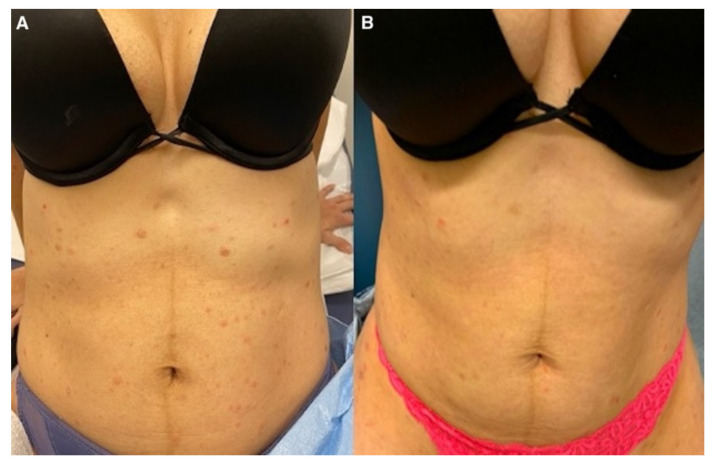
(**A**) Abdomen before medical management; (**B**) after azithromycin initiation.

**Figure 5:**
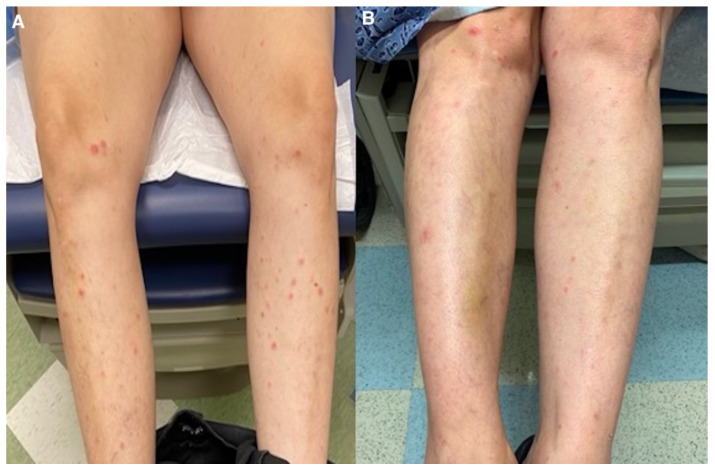
(**A**) Lower extremities before medical management; (**B**) after azithromycin initiation.

Treatment with azithromycin 500 mg on day 1 followed by 250 mg daily until day 5 was initiated. This regimen was repeated every two weeks. The patient noticed slight improvement after 1 week of treatment (Figure 4 and Figure 5). At 5 months follow-up, with continued azithromycin every 2 weeks in addition to triamcinolone 0.1% cream daily, she had marked improvement of the rash with complete resolution of the vast majority of lesions and scattered postinflammatory hyper- and hypopigmentation. 

Based on the clinical, histopathologic findings and response to treatment, the patient was diagnosed with PLEVA possibly developing as a complication of a COVID-19 infection.

## 3. Discussion

PLEVA manifests as an acute eruption of small, erythematous to brown or purpuric macules that rapidly develop into papules with overlying hemorrhagic crust. The eruption, often involving the trunk and extremities, is polymorphic with lesions at different stages of evolution, varying in severity and temporal development. While it is generally asymptomatic, burning or pruritus may be present. The lesions may also spontaneously resolve with secondary postinflammatory hyper- or hypopigmentation [1,2].

The pathogenesis of PLEVA is unclear. Current theories include infectious etiologies with pathogenic culprits including human immunodeficiency virus, human herpesvirus 7, varicella zoster virus, Epstein–Barr virus, cytomegalovirus, and parvovirus B19 [2,3]. Several reports have described unique presentations of PLEVA characterized by a CD30 (+) component and clonal TCR− γ gene rearrangement in the setting of viral infection [4,5,6,7,8]. Our case similarly demonstrated a CD30 (+) infiltrate and a positive TCR− γ gene rearrangement study. Recently, 11 patients with PLEVA induced by a COVID-19 infection [9,10] and 7 by the COVID-19 vaccine [11,12,13,14,15,16,17] have been reported in the literature (Table 1). In the setting of a recent COVID-19 infection, these findings suggest that COVID-19 should likely be added to the list of possible viral agents capable of inducing PLEVA. 

On the contrary, some consider PLEVA as a type of cutaneous lymphoproliferative disorder showing overlapping features with mycosis fungoides (MF) and lymphomatoid papulosis (LyP) [7]. In one study, a clonal TCR− γ gene rearrangement was reported in 65% of cases [8]. Additionally, while generally considered an inflammatory condition, some authors have noted various patients with pityriasis lichenoides preceding diagnoses of classic mycosis fungoides or Hodgkin lymphoma and other patients with self-healing lesions resembling PLC coexisting with mycosis fungoides. These authors postulate that the so-called “lymphomatoid” pityriasis lichenoides may exist on a spectrum or continuum with both MF and LyP [18,19].

Histopathological evaluation of PLEVA usually reveals epidermal spongiosis, mild to moderate acanthosis, parakeratosis, dyskeratosis, and occasional vacuolar degeneration with intraepidermal vesicles. The dermis usually shows edema, a dense and diffuse inflammatory infiltrate composed of CD8 (+) T-lymphocytes along the basal layer of the epidermis with prominent lymphocytic exocytosis into the epidermis. Advanced findings usually encountered in febrile ulceronecrotic Mucha−Habermann disease may show an extension of the inflammatory infiltrate into the epidermis, extravasation of erythrocytes, widespread epidermal necrosis, and nuclear debris in necrotic areas [1,2]. Histopathological examination of our patient showed features such as vacuolar degeneration of the basal layer with necrotic keratinocytes and dense deep periglandular lymphocytic infiltration, which have been consistently described in COVID-19-related dermatosis [20].

The major histologic differential diagnosis to consider is lymphomatoid papulosis (LyP). Generally, the degree of cytologic atypia in lesions of LyP is more severe and extensive than would be expected in PLEVA. CD30 has been considered by some authors as one of the main differential diagnostic criteria between LyP and PLEVA. However, similar to our patient, cases of a unique CD30+ PLEVA variant have been published in case reports and series. Although some have suggested that the absence of large CD30 (+) cells in the dermis may point towards a histologic diagnosis of PLEVA, the distinction between PLEVA and LyP in some cases is difficult, and the diagnosis may require extensive clinicopathologic correlation and follow-up [7,8].

Overall, PLEVA is very difficult to treat because of its unknown etiology and unpredictable course. Currently, there is no standard treatment for this condition, but a combination therapy is frequently considered the best approach [21]. Treatment options include phototherapy, oral antibiotics including macrolides and tetracyclines, topical corticosteroids, and antihistamines [21,22]. Azithromycin has been proven as a valid treatment and effective alternative when patients are unresponsive to the first line of treatment or when phototherapy is unavailable [23]. Systemic medications such as corticosteroids, methotrexate, cyclosporine, and retinoids may be warranted in severe manifestations of PLEVA unresponsive to conventional treatment [21,24].

## 4. Conclusions

We report an atypical case of PLEVA presenting as a complication of a COVID-19 infection. While histologically typical features were present, unusual features masking more typical histologic findings included the presence of a predominantly intraepidermal CD30+ infiltrate and a positive clonal TCR− γ gene rearrangement study. Similar findings have been seen in association with other virally induced cases of PLEVA. In this patient, the clinicopathologic correlation and response to treatment was paramount in confirming the diagnosis of PLEVA. Awareness of these reactions following a COVID-19 infection, accompanied by a comprehensive histopathologic evaluation of skin biopsies, should be completed to determine if PLEVA is directly related or not to the infection. The current level of evidence on this association is limited and based solely on case reports and case series. Further studies with systematic dermatological examination of patients with proven COVID-19 are needed to establish a formal association between infection and the development of PLEVA.

## Figures and Tables

**Table 1:** Summary of a COVID-19 infection and vaccine-induced PLEVA.

Reference	Infection/Vaccine	Histopathology	Cutaneous Lesions
Durusu et al. [10]	COVID-19 infection	Hyperkeratosis; irregular acanthosis; focal spongiosis; lymphocytic exocytosis in the epidermis; and band-like lymphomonocytic infiltrate and melanophages in the superficial dermis.	Erythematous to purple lichenoid papules and plaques and hemorrhagic crusts.
Gianotti et al. [9]	COVID-19 infection	Dermal lymphocytic infiltrate; interstitial eosinophils; diffuse interface dermatitis; and scattered necrosis of keratinocytes.	Erythematous and purpuric hemorrhagic papules with crusts.
Sechi et al. [14]	2nd dose Pfizer vaccine	Focal epidermal ulceration; spongiosis; parakeratosis; and interface inflammation within a wedge-shaped dermal inflammatory cell infiltrate.	Scattered, nonfolliculocentric papules with erythematous, raised borders and an eroded center, covered by a hemorrhagic crust.
Sernicola et al. [12]	1st dose Pfizer vaccine	Hyperkeratosis; epidermal hyperplasia; diffuse spongiosis with a foci of mixed, lympho-monocytic infiltrates; Langerhans cells; granulocytes; and a dense, polymorphic inflammatory infiltrate in the dermis.	Erythematous-pinkish papular lesions partially covered by sero-hematic crusts.
Palmén et al. [11]	1st dose Pfizer vaccine	N/A	Erythematous, ulcerative, and crusting lesions.
Dawoud et al. [13]	1st dose Pfizer vaccine	Parakeratosis; moderate spongiosis and focal vacuolar alteration of the basal cell layer; mild edema; extravasated red cells; and superficial and deep dermal perivascular, lymphocytic infiltrate.	Erythematous and purpuric hemorrhagic papules with crusts.
Filippi et al. [17]	1st dose AstraZeneca vaccine	Parakeratosis; mild spongiosis; wedge-shaped perivascular lymphocytic infiltrate; and apoptotic keratinocytes and extravasated erythrocytes in the papillary dermis.	Erythematous and erythematous-crusted papules.
